# Fusarium Tropical Race 4 in Latin America and the Caribbean: status and global research advances towards disease management

**DOI:** 10.3389/fpls.2024.1397617

**Published:** 2024-07-16

**Authors:** Thayne Munhoz, Jorge Vargas, Luiz Teixeira, Charles Staver, Miguel Dita

**Affiliations:** ^1^ Laboratório de Microbiologia Ambiental, Embrapa Meio Ambiente, Jaguariúna, Brazil; ^2^ Biodiversity for Food and Agriculture, 2 Centro Internacional de Agricultura Tropical, Cali, Colombia; ^3^ Centro de Solos e Pesquisas de Fertilizantes, Instituto Agronômico, Campinas, Brazil; ^4^ Facultad de Agronomía, Universidad Veracruzana, Xalapa, Mexico; ^5^ Biodiversity for Food and Agriculture, Bioversity International, Cali, Colombia

**Keywords:** banana, tropical race 4, *Fusarium oxysporum f.* sp. *cubense*, integrated disease management, *Musa spp*

## Abstract

Fusarium wilt of banana (FWB), caused by the soil-borne fungus *Fusarium oxysporum* f. sp. *cubense* (Foc), poses an undeniable threat to global banana production. This disease has intensified in recent years, with the tropical race 4 (TR4) strain spreading rapidly. Since 2018, the number of affected countries has increased from 16 to 23, presenting a significant challenge to researchers, producers, and National Plant Protection Organizations (NPPOs) worldwide. The potential impact of TR4 in Latin America and the Caribbean (LAC) is particularly concerning. This region boasts seven of the top ten banana-exporting countries, and bananas and plantains are crucial for food security and income generation. In Colombia, where TR4 was detected in 2019, the disease has already spread from La Guajira to Magdalena, and it is currently affecting 20 large commercial export farms. In Peru, the disease was detected in 2021 and although still restricted to the northern region, flood irrigation and heavy rains associated with the Yaku cyclone, boosted pathogen spread, and more than 400 small organic banana farmers are currently affected. In Venezuela, TR4 detection occurred in 2023, with plantations across three states and five municipalities now affected. Worryingly, TR4 has also been confirmed in plantains, a staple food in the region. Current national responses in LAC primarily rely on preventive and reactive measures: preventing initial incursions and containing outbreaks to avoid further spread. However, the disease’s relentless progression suggests that its eventual presence in all banana-producing areas is likely. Therefore, exploring alternative management approaches beyond pathogen exclusion becomes crucial, both in affected and disease-free regions. This paper examines the current spread of TR4, focusing on epidemiological aspects and recent research-based management options. Key epidemiological features were highlighted, drawing practical examples from various scales (plots to landscapes) and utilizing experiences from LAC’s fight against TR4. The paper also reviews field-tested approaches in biosecurity, biological control, resistant varieties, soil health, and integrated disease management, acknowledging the specific challenges faced by smallholder settings. In each section research initiatives were analyzed, identifying gaps, and proposing directions to minimize TR4 impact and accelerate the development of sustainable solutions for managing this devastating disease.

## Introduction

Bananas are among the most important global export fruit, but exports account for only 16% (about 25 million metric tons) of world production (Loeillet and Dawson, 2023). Smallholder farmers and domestic urban markets consume the remaining 84%, highlighting bananas’ importance for food security and income.

Latin America was responsible for 60% of the 21 million tons exported bananas in 2022 (Loeillet and Dawson, 2023). Eight countries (Ecuador, Colombia, Costa Rica, Guatemala, Honduras, Panama, Mexico, and the Dominican Republic) from Latin America and the Caribbean (LAC) are ranked among the top ten global exporters (Loeillet and Dawson, 2023). Ecuador, Dominican Republic and Peru are the top three organic banana exporters (Dawson, 2023). These exports are largely from Cavendish (AAA) bananas grown in perennial monocropping systems on medium- and large-scale farms, although small growers are important in organic export bananas. Cavendish is also widely grown for national urban markets and regional trade not only in Latin America, but also in Asia and Africa, making up nearly 50% of global production (Dita et al., 2013a; Loeillet and Dawson, 2023).

LAC also produces many other banana varieties in diverse systems, from annually replanted monocrops to mixed agroforestry with coffee and cocoa. Plantains (AAB), hardy cooking bananas (Bluggoe, ABB), Gros Michel (AAA), Red banana (AAA), South Pacific plantains (AAB), Prata (Pomme, AAB), Silk (AAB) and Pisang Mas (AA) are grown both for income and food security (Dita et al., 2013a). These varieties make up 32% of production (Loeillet and Dawson, 2023).

Fusarium wilt of banana (FWB) caused by the soil-borne pathogen *Fusarium oxysporum* f. sp. *cubense* (Foc), the topic of this paper was historically a major problem in LAC, ranked among the most important diseases in agriculture by Simmonds in 1966. The emergence and spread of the tropical race 4 strain (TR4) is generating new and growing concern.

**Figure 1:**
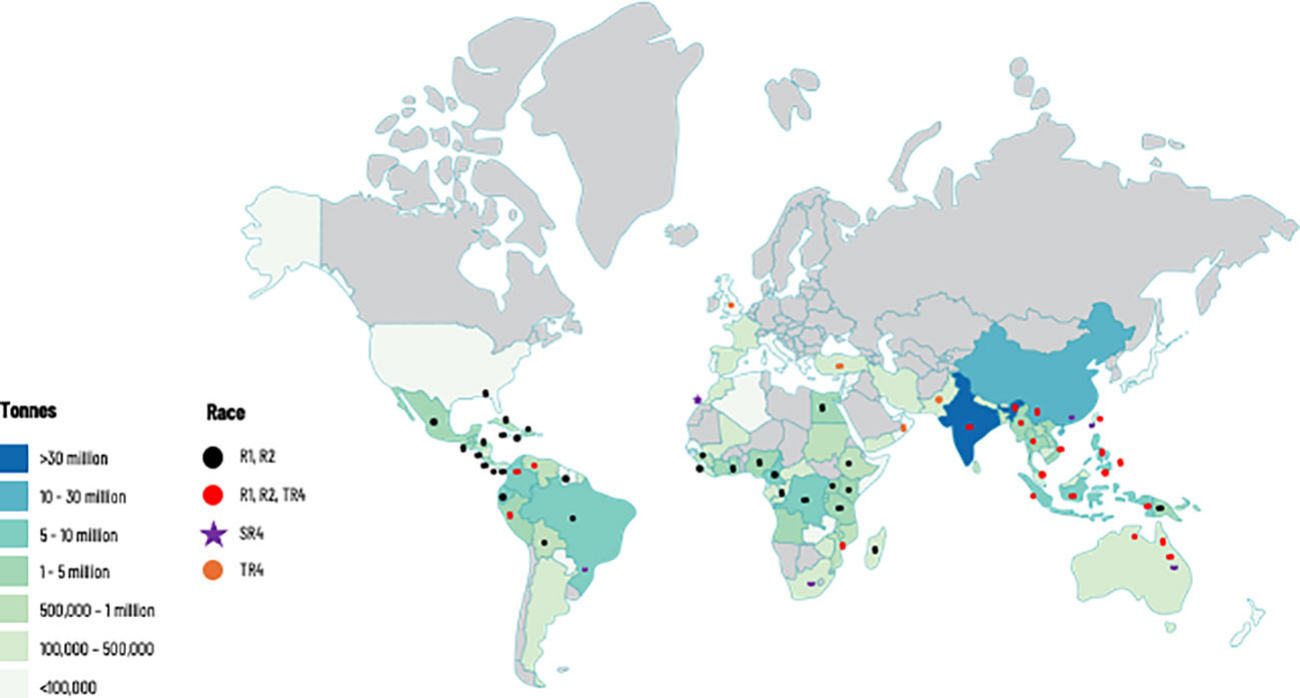
Global production of banana and distribution of races of *Fusarium oxysporum* f. sp. *cubense* (Foc), causal agent of Fusarium wilt of banana. This map considers producing countries with presence or absence of a given race of Foc and does not represent banana-producing areas by countries. R1: Race 1; R2: Race 2; SR4: Subtropical race 4; TR4: Tropical race 4. Races 1 (R1) and 2 (R2) are widely distributed in banana producing countries affecting local varieties (see introduction for more details). Subtropical Race 4 (SR4) corresponds to Foc populations present in subtropical producing areas in Australia, Brazil, Canary Islands, China, South Africa, and Taiwan, causing intermittent yield losses in Cavendish cultivars. Tropical race 4 is the most virulent strain and considered the major threat for the global banana industry. For more information on Foc races see **Table 1**.

The spread of TR4 to major banana-growing countries in the Americas was considered only a matter of time (Ploetz, 2015). In 2019, TR4 was reported in Colombia (García-Bastidas et al., 2019), in 2021 in Peru (Acuña et al., 2022) and in 2023 in Venezuela (Mejías et al., 2023). With the arrival in LAC and its alarming global spread (**Figure 1**), TR4 can be considered a pandemic. Cavendish, the mainstay of the export sector, is extremely susceptible along with many local varieties. The epidemics caused by Foc race 1 (R1) in the last century were addressed in the export industry by replacing the Gros Michel variety, highly susceptible to R1, with the R1-resistant Cavendish cultivar. The export banana sector became Cavendish-driven and FWB research was abandoned by the transnational banana companies with only a few countries continuing research in their national programs. However, strains of R1 and race 2 (R2) continued to spread among farms producing susceptible varieties for national markets (Dita et al., 2013a; 2018).

Efforts by National Plant Protection Organizations (NPPOs) to contain R1 and R2 in production for national consumption have been minimal. Farmers, unaware of FWB symptoms, pathways of spread, and persistence in the soil, have adapted to the increasing area of Foc-contaminated lands either by changing crops, switching to R1 and R2-resistant varieties, or seeking out Foc-free soils (Dita et al., 2018). Even today, growers continue to spread Foc R1 and R2 into uninfected fields in LAC (Dita et al., 2018). Local markets still prefer local R1-susceptible varieties like Gros Michel, South Pacific plantains, Prata (Pomme, AAB) and Silk which have no FWB-resistant substitutes with similar production, commercial and eating properties.

The menace of FWB derives from its persistence as pathogen spores in the soil for up to 30 years in the absence of banana. Spore survival was considered the main mechanism but multiplication in other plant hosts also contributes to spore persistence (Pegg et al., 2019). Once soils are TR4-contaminated, susceptible varieties cannot be successfully replanted again (Stover, 1990). The spread of the disease, while difficult to prevent, is slow depending on any vector of contaminated soil and contaminated planting material (Pegg et al., 2019). The newly affected zones will shift over time, but it seems unlikely that consumers across the globe either in producing or importing countries will witness the disappearance of bananas, since uncontaminated soils are still abundant. On the medium term, bananas will continue to be among the lowest-cost fruit for consumers.

Recent overview articles on the threat of plant pathogen invasion have cited the case TR4 (Fones et al., 2020; Drenth and Kema, 2021; Ristaino et al., 2021) to highlight important research and biosecurity priorities to address food security and income losses. The threat that TR4 represents for the global banana sector has been addressed in recent scientific articles (Drenth and Kema, 2021; Silva et al., 2023) and research efforts are increasing. Review articles have summarized specific aspects of TR4, such as the potential of biological control (Bubici et al., 2019); epidemiology and management options (Dita et al., 2018; Pegg et al., 2019).

Our objective in this article is to describe the current spread of TR4 in LAC and review recent global research advances for their relevance to improve research programs and TR4 management in LAC. In synthesis, we build on our field experience with both the ongoing spread of R1 and R2 and the recent TR4 outbreaks and spread in LAC to propose that locally FWB is having and will continue to have a devastating impact well into the future. After a brief overview of the disease and pathogen population biology, we summarize field experiences of TR4 outbreaks in LAC and factors complicating effective responses. We then address two dimensions of disease management starting with recent advances in disease exclusion, detection, and containment. This is followed by an examination of host resistance to TR4, biological control, soil health and integrated disease and cropping system management. Based on these sections, we identify gaps and propose priority research and actions to minimize the pathogen spread to free TR4-free areas and maintain banana production where the pathogen is already present.

## The disease

**Figure 2:**
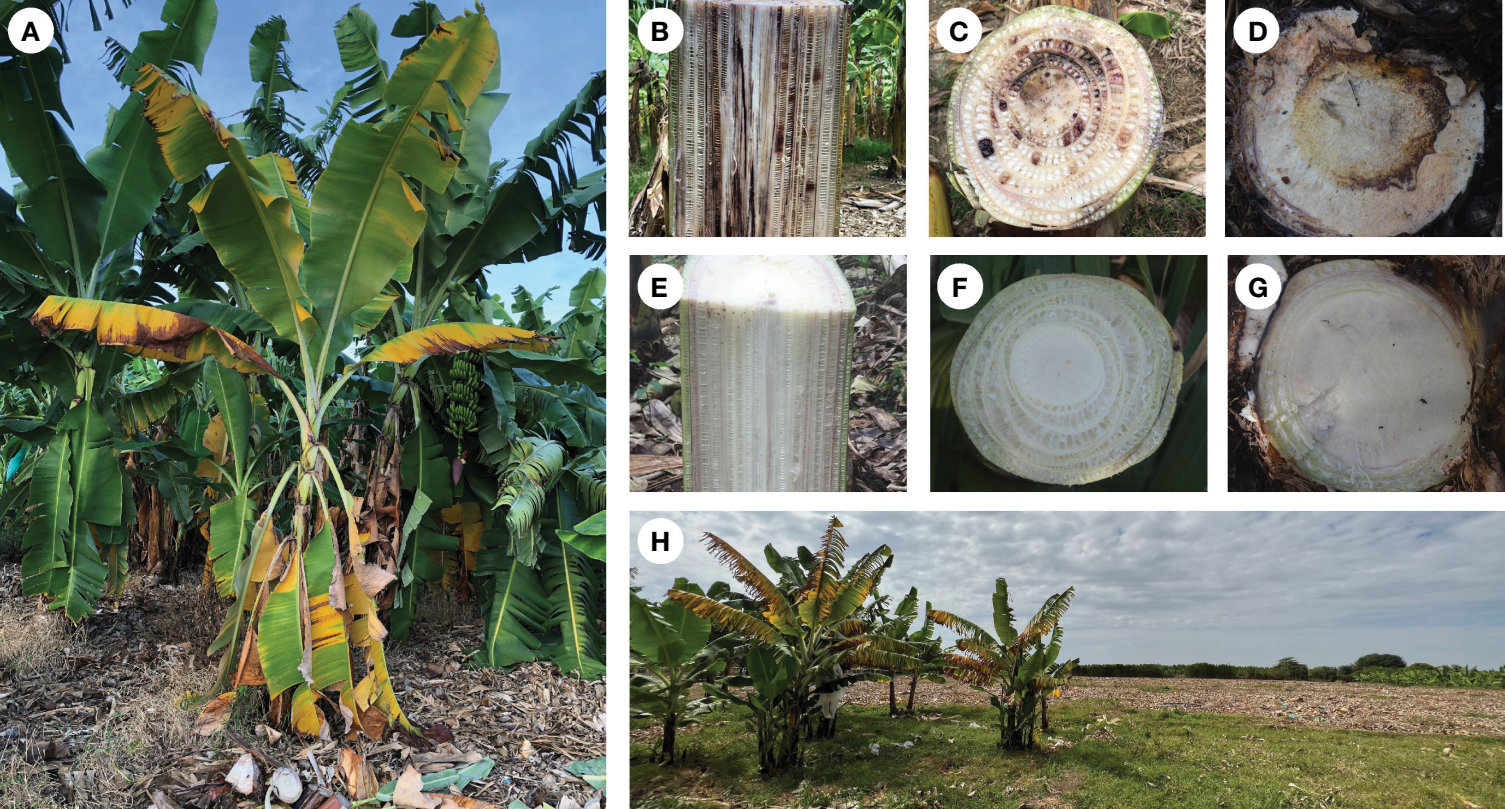
Symptoms of Fusarium wilt of banana (FWB), tropical race 4 (TR4) in Cavendish cultivar. Plant showing typical external symptoms **(A)**. Longitudinal **(B)** and transversal cuts **(C)** showing internal symptoms in the pseudostem. Transversal cuts showing internal symptoms in the rhizome **(D)**. Longitudinal **(E)** and transversal cuts of pseudostem **(F)** and rhizome **(G)** of healthy banana plants. Banana field severely affected by TR4 in Peru approximately one and a half year after the pathogen incursion **(H)**.

The arrival of TR4 to LAC has demonstrated that more efforts are needed to understand this disease both at farm and at landscape levels. In short, Foc is a soil-borne pathogen that infects banana plants through secondary roots. Once inside the plant the pathogen colonizes and destroys the vascular system causing external symptoms which start as yellowing and wilting from older to younger leaves (**Figure 2A**) and internal symptoms characterized by necrosis on the vascular system (**Figures 2B, C**), and rhizome discoloration (**Figure 2D**). Infected plants also may show delayed development and abnormal leaf growth. A comparison of affected with healthy plants (**Figures 2E–G**) illustrates that Foc deprives affected plants of water and nutrient uptake preventing normal fruit production and causing plant death. Once a FWB epidemic starts if management measures are not implemented opportunely, widespread destruction may occur rapidly (**Figure 2H**).

## The pathogens

Ploetz, 2006, in the article “Fusarium Wilt of Banana Is Caused by Several Pathogens Referred to as *Fusarium oxysporum* f. sp. *cubense*” reflected on the huge genetic diversity of Foc drawing on its several independent evolutionary origins. The genetic diversity of Foc has been extensively documented recently (Mostert et al., 2017, 2022; Maryani et al., 2019; Batista et al., 2022; Ma et al., 2023) and it is not the scope of this review deepen on Foc population biology. However, with the identification of TR4 and the progress of genome sequencing, more information on Foc populations has been generated, bringing more complexity and in some cases confusion to the current racial structure of this pathosystem.

The utilization of Vegetative Compatibility Groups (VCGs) and SIX (Secreted In Xylem) genes as tools for discriminating Foc populations and for diagnostic complementation poses challenges in deciphering connections between VCGs, SIX genes, races and diagnostic methodologies. VCG refers to the ability of two isolates to undergo vegetative hyphal fusion (heterokaryosis) (Puhalla, 1985). The members of a particular VCG are clonal lineages and share similar pathological, physiological, and biological attributes (Caten and Jinks, 1966). SIX genes refer to a specific set of genes found in *F. oxysporum*, particularly those strains that cause Fusarium wilt, such as Foc (Rep et al., 2002, 2004). These genes encode proteins that are secreted by the fungus into the xylem vessels of infected plants and can be used as molecular markers to identify and differentiate between pathogenic strains or even races (Czislowski et al., 2018; Carvalhais et al., 2019). On the other hand, races refer to genetically distinct groups within a single species of pathogen that differ in their ability to infect specific varieties or cultivars of a host plant (Anderson et al., 2010). These differences in pathogenicity are often due to variations in the pathogen’s effector genes (such as SIX genes), which interact with the host plant’s resistance genes.

In the case of the Foc-banana interaction race discrimination is also based according to the ability of certain strains to infect Cavendish depending on the environmental (subtropics and/or tropics) conditions (**Table 1**), which may also complicate the understanding of Foc diversity and disease epidemiology.

**Table 1:** Racial structure of *Fusarium oxysporum* f. sp. *cubense* (Foc), banana varieties used as reference and associated Vegetative Compatibility Groups (VCGs).

^a^ Race	^b^ Reference varieties (Genome)	Associated VCGs
R1	Gros Michel (AAA), Silk (AAB) and Pisang Awak (ABB)	All **VCGs**, except 01213/16
R2	Bluggoe (ABB)	All **VCGs**, except 01213/16
SR4^c^	Cavendish (AAA)	0120, 0121, 0122, 0126, 0129, 01210, 01211, 01215
TR4^d^	Cavendish (AAA), Gros Michel (AAA), Silk (AAB) and Pisang Awak (ABB)	01213/16

a Races in Fusarium oxysporum f. sp. cubense (Foc) here refers to groups of pathogenic strains with a differential reaction on a set of banana varieties. The racial structure for Foc is imperfect. For instance, in Cuba, R1 strains from Silk bananas have been reported affecting Bluggoe bananas (Martínez de la Parte, 2023). Some, of these R1 and R2 strains have been recently proposed as new and different species (Maryani et al., 2019). Foc populations are very diverse and new VCGs (Batista et al., 2022; Mostert et al., 2022) and even species (Maryani et al., 2019) have even been proposed. Racial differentiation of Foc is presented here only for the purpose of illustrating some frequently used terminology and the challenges of associating races to specific VCGs.

b Names of variety may differ depending upon country and production region; only varieties used as references for Foc races were listed here.

c Subtropical race 4 (SR4) are Foc populations of at least eight VCGs able to infect Cavendish only under subtropical conditions. Strains of these populations are found affecting R1 and R2 -susceptible varieties under tropical conditions and are therefore named as R1.

d Tropical race 4 (TR4) is a clonal population of VCG01213/16 able to infect Cavendish under both tropical subtropical conditions. TR4 also affects many other varieties, and it is currently considered the most virulent Foc strain. This strain has been proposed as a new species by Maryani et al. (2019) but contested by Torres Bedoya et al. (2021).

Firstly, the racial structure of Foc is not determined based on pathogen virulence and banana resistance genes, as is commonly observed in many plant diseases (Anderson et al., 2010). Secondly, the scientific foundation of VCG analysis is not inherently linked to pathogenicity or virulence (Puhalla, 1985). Therefore, establishing associations between VCGs and specific races, particularly for Foc R1 and Foc R2 populations, may be questionable (**Table 1**). Thirdly, there remains a dearth of knowledge regarding SIX genes and their role in pathogenicity or virulence across global Foc populations and banana genotypes (Fraser‐Smith et al., 2014; Czislowski et al., 2018, 2021; Ma et al., 2023). Consequently, linking Foc races, VCGs, SIX genes, and/or single PCR analysis, although feasible for selected strains (Fraser‐Smith et al., 2014; Carvalhais et al., 2019), remains challenging given the extensive diversity of Foc populations worldwide. These challenges are further compounded by recent reports of new, yet uncategorized VCGs reported in Asia (Mostert et al., 2022) and in Brazil (Batista et al., 2022).

The current understanding of the race structure of Foc, reference varieties, and VCGs highlights the complexity involved in associating genetically unrelated tools to understand and differentiate Foc populations (**Table 1**). For example, while TR4 is associated with a unique VCG (01213/16), strains of Subtropical Race 4 (SR4), which primarily affect Cavendish under subtropical conditions, are distributed among at least eight different VCGs (**Table 1**). As the name suggests, SR4 strains do not affect Cavendish in tropical regions but are widely distributed and impact Gros Michel (as well as numerous other local varieties) in tropical areas, where they are classified as R1. This distinction is particularly evident for strains belonging to VCG 0120/15, which can affect Cavendish in subtropical regions such as Australia, Brazil, the Canary Islands, and South Africa (Dita et al., 2020), but not in tropical regions. Consequently, the categorization of a given Foc population as R1 in the tropics and as SR4 in subtropical regions is primarily based on the imperfect Foc racial structure. Whether the global Foc populations considered as R1 in the tropics and as SR4 in the subtropics harbor the same set of pathogenicity/virulence/effector genes is a question that needs to be addressed. Extensive monitoring of occurring Foc populations in LAC is required, but capacities to do high throughput genotyping are not available in most banana-producing countries.

## Incursions and spread of TR4 in Latin American and the Caribbean

The identification of mechanisms facilitating the introduction of TR4 into a country could significantly enhance the development of a more robust risk assessment scale and enable the refinement of exclusion tactics at both borders and on-farm levels. However, this endeavor has proven elusive thus far globally and in LAC.

In a study conducted in 2014, Scheerer et al. (2018) projected that Colombia would be among the first countries to experience a TR4 incursion within ten years from the study’s date, with Peru following 5-10 years thereafter. Venezuela was not included in the study due to its limited extent of export banana production.

Since the first outbreak of TR4 was reported in Colombia in August 2019 (Garcia-Bastidas et al., 2019), the disease has rapidly spread. Initially affecting three farms in La Guajira, it has currently reached 20 farms, with 11 in La Guajira and nine in Magdalena, a department located more than 150 km away from the initial TR4 outbreaks. It is estimated that more than 300 hectares have been eradicated and are no longer in production due to restrictions on new planting in affected areas. No official information on the number of TR4 cases by farm in Colombia has been officially released so far.

In Peru, the spread of TR4 is monitored through statistics on disease detection and eradication outbreaks (cases) carried out by the NPPO (SENASA). SENASA estimated that more than 400 outbreaks have been eradicated in early 2024.

For the 23 countries reporting TR4 outbreaks globally, very little country-specific information on current spread is available. Australia is an exception reporting the number of infected mats (Pegg et al., 2021). For the three countries in Latin America, as the TR4 epidemics have accelerated, the challenges to simply tracking disease spread have multiplied.

While Colombia reports affected farms and Peru numbers of TR4 outbreaks, Venezuela has reported number of municipalities and states affected by TR4. According to the NPPO (INSAI), TR4 is impacting banana plantations across three states and five municipalities (INSAI, 2023a). Unlike Colombia and Peru, where TR4 has been so far detected only in Cavendish bananas, in Venezuela, the disease has also been confirmed for the first-time affecting plantains (INSAI, 2023b), a staple food in many countries across the LAC region (Dita et al., 2013a).

Evidence suggests lag times of up to 3-4 years before the initial outbreak is detected. While the official sequence of outbreak detection for Latin America is Colombia, Peru, and Venezuela, this may not necessarily reflect the actual sequence of pathogen introduction. In addition, genomic analyses suggested independent TR4 incursions in the Americas as TR4 strains from Peru were clustered together but separated from those from Colombia (Leiva et al., 2022). Unfortunately, no genomic information on the TR4 strain from Venezuela is yet available.

As a soil-borne pathogen, Foc can be disperse by animals, insects, birds, shoes, machinery, tools and trucks. In addition, planting material and water have proved to be very effective on disseminating Foc (Dita et al., 2018; Pegg et al., 2019).

Scheerer et al. (2018) projected in-country spread of TR4 based on three factors – strength of national quarantine measures, importance of Cavendish and importance of banana in research and development policy – in percent of banana area infected. However, they recognized the role of biophysical factors, which they could not capture across the 29 countries in the study. This has been evident in the spread after initial outbreaks in LAC. For instance, the increase in TR4 cases in Colombia in 2022 was associated to flooding (ICA, 2022). Similarly, the monthly count of TR4 outbreaks in Peru increased from nine to 50 from April to July 2023, likely due to flooding triggered by the Yaku cyclone, which impacted TR4-affected areas in March 2023. Given that TR4 is present in production systems for domestic consumption in Venezuela, efforts to track and contain its spread seems to face nearly insuperable challenges.

Local features of banana production and marketing, weather, farm size and the diversity of land use in areas where bananas are grown will play varying roles in how the disease spreads further. In the export banana zone of Peru, some affected small growers (banana farms average 1 hectare) have already converted to rice and maize production. This conversion increases pathogen spread risks due to the movement of contaminated soil into irrigation canals and on the footwear of workers since their immediate neighbors continue to produce banana in contiguous areas. In Colombia, 20 affected farms in two states represent an expanding disease front accelerating the spread to farms for local production and new export areas.

Despite the absence of reports indicating a secondary introduction of TR4 into the same country, such occurrences cannot be ruled out. The prospect of new introductions into either the same or different zones would pose additional challenges to containment efforts.

Dita et al. (2018) and Pegg et al. (2019) summarized epidemiological understanding of FWB. However, the arrival of TR4 to LAC has demonstrated that more efforts are needed to understand this disease at different levels. From the disease management perspective, these spreading factors can be categorized into long-distances (planting material, soil, machinery, irrigation channels, rivers) and short-distances (replanting, irrigation water, animals, farmers, harvesting crews etc.). Identifying and managing these factors (when possible) reduces pathogen spread on farm, from farm-to-farm and, consequently, at landscape levels. However, managing some dispersal factors could be impractical. For instance, all the banana farms in Northern Peru rely on flood irrigation with many farms sharing the same canals. Once water sources are Foc-contaminated, returning to Foc-free water is nearly impossible. Water through extreme weather seems to be one of the most important factors to disseminate Foc, and other banana diseases, such as Moko (*Ralstonia solanacearum*, race 2) and bacteria soft rot (*Pectobacterium carotovorum* subsp. *carotovorum*/*Dickeya paradisiaca*). In LAC every year many hurricanes, tropical storms or other severe climate events affect most of banana producing areas in the region.

## Management options in the context of Latin American and the Caribbean

This section highlights key learnings from research and practical experience on reducing TR4 spread and sustaining banana production in fields already infected with TR4. We focus on key components of an integrated approach for TR4-compatible banana cropping systems: Exclusion, Early detection, and plant destruction; Biosecurity; Resistant varieties; Biocontrol and Integrated soil health-oriented practices and cropping systems. By reviewing recent advancements in these areas globally, we aim to identify research priorities and address major challenges facing its implementation for LAC.

### Exclusion, early detection, and plant destruction

Exclusion as a strategy is based on phytosanitary surveillance and implementation of biosecurity measures at the country, region, farm, or field level. Country-level exclusion is the responsibility of the NPPOs, which in the case of Foc TR4 have made efforts at ports and airports, and land borders. Container and vehicle washing, baggage scanning, canine inspection, the establishment of footbath for footwear disinfection, prohibition of movement of plant material, as well as sensitization of travelers through outreach materials and alerts have been recommended and implemented to varying degrees. These measures were increased in LAC, mainly after the disease was reported in Colombia. Container disinfection, for example, is mandatory in Colombia according to the resolution 00002081 issued by the Instituto Colombiano Agropecuario (ICA, 2024). However, the rigor of implementation varies according to the country. At many land borders in LAC, these measures are difficult to implement or of questionable effectiveness, due to the existence of numerous informal crossings. In that scenario, implementing biosecurity at the farm level, which is the responsibility of the producer, is essential and will be addressed later.

Early detection and plant destruction of mats showing FWB symptoms is key to successful pathogen containment, and overall disease management. The main objective behind this practice is to reduce the inoculum build up. The earlier infected plants are detected, the greater the chances of success in containing the disease. For this, it is essential to carry out frequent monitoring of plantations to detect suspicious plants (Dita et al., 2018). Although FWB is an “old disease” (Bancroft, 1876), in many places farmers are not familiar with the disease symptoms. After the introduction of Cavendish in the 1960s, in many banana production areas in LAC, FWB became a forgotten disease. However, other vascular diseases such as Moko (*Ralstonia solanacearum* race 2) and bacterial soft rot (*Pectobacterium carotovorum*/*Dickeya paradisiaca*), which cause symptoms that can be confused with FWB, continued to be present in many of these production areas (Blomme et al., 2017). In all three cases of TR4 in LAC, at least one of those bacterial diseases, primarily bacterial soft rot, has been present, both alone and in disease complexes with TR4. For example, in Venezuela a fungal-bacterial disease complex called banana wilt (BW) was reported in 2022 (Olivares et al., 2022). Coincidentally, the BW disease complex was reported in the state of Aragua, in the same areas where TR4 was later detected (Mejías et al., 2023).

Technicians and farmers must not only be trained to detect FWB, but also to discriminate it from these bacterial diseases and identify the possible co-occurrence of disease complexes. In areas beyond export banana production, Foc R1 and R2 are widely spread affecting local varieties and causing symptoms similar to TR4. This is an additional challenge to early detection of TR4 as the pathogen may appear in local varieties instead of Cavendish, as occurred in the Mayotte Islands (Aguayo et al., 2021).

The responsibility for monitoring prior to the first outbreak is put fully on the NPPOs which face numerous obstacles. Staver et al. (2023) found that some growers in Peru had been trained in FWB disease symptoms. However, members of the harvesting crews, day laborers, certification crews who move from farm to farm each day or are present weekly in plantations were not trained. Training all participants in production and packing offers a real-time surveillance opportunity for disease detection which is currently weakly developed.

**Figure 3:**
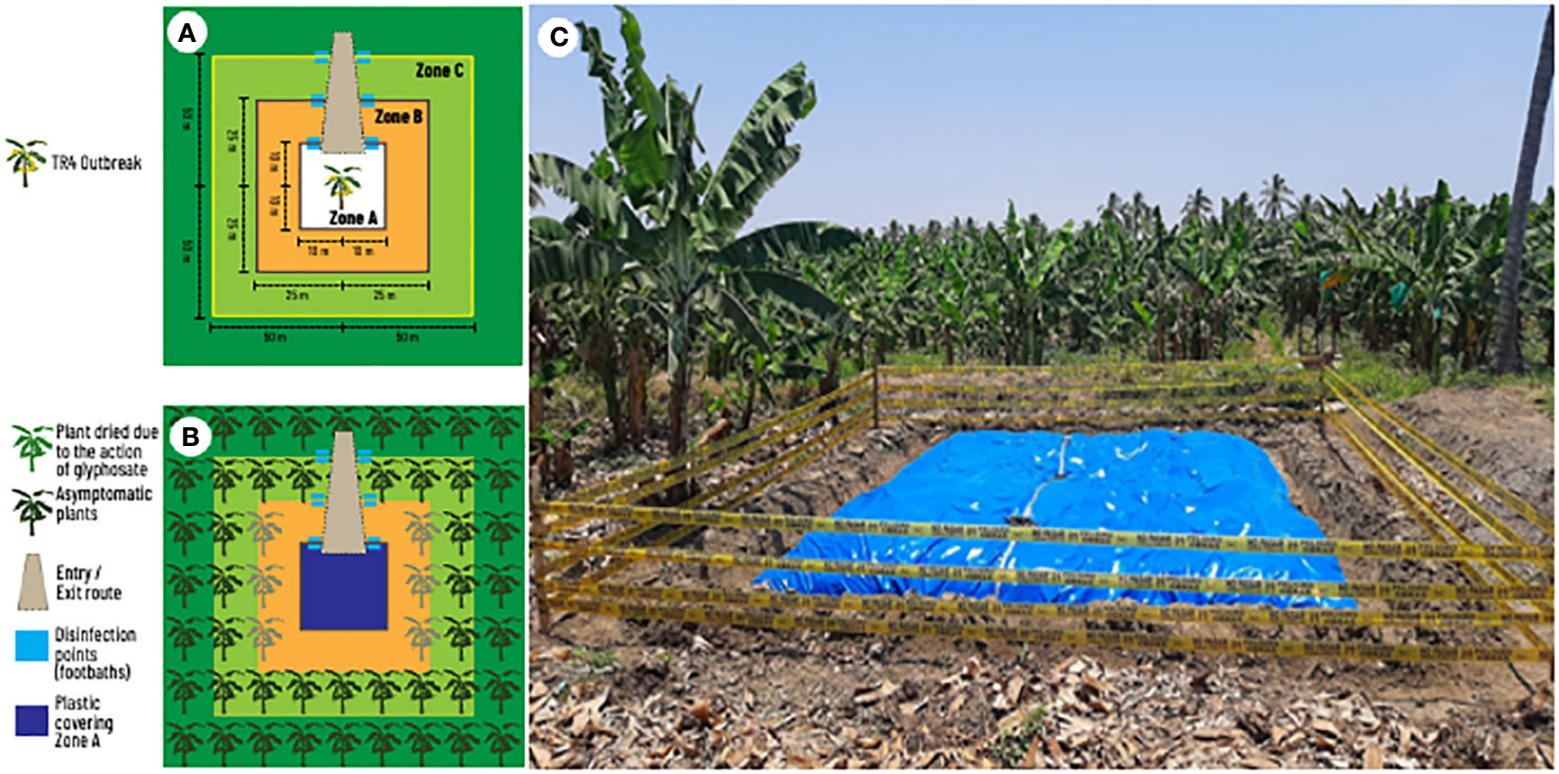
Components of field eradication of an early outbreak of tropical race 4 (TR4) of *Fusarium oxysporum* f. sp. cubense in a banana monoculture. **(A)** Distances and zones from infected plants. **(B)** Location and quarantine measures in an eradication area. **(C)** Eradication area previously affected by TR4 in Peru showing zone A covered by plastic.

Once the disease is detected and new outbreaks begin to appear, a discussion begins regarding the size of the area to be destroyed. The contingency plan developed by the Organismo Internacional Regional de Sanidad Agropecuaria - OIRSA (Dita et al., 2013b) has served as a guide for most NPPOs in LAC. The OIRSA plan proposes three zones with different sizes to contain a hypothetic first TR4 outbreak in LAC (**Figures 3A, B**). A plant destruction zone (Zone A: 100 m^2^), a buffer zone (Zone B: 2500 m^2^) and an observation zone (Zone C: 10000 m^2^) were established considering a large monocrop plantation. In zone A, plants should be mechanically destroyed, uprooted followed by urea application and covering with a plastic tarp. In zone B, plants should be injected with glyphosate to die intact. In zone C, plants are not destroyed and are continuously monitored. For a plantation with 2000 plant per hectare, an estimate of at least 500 plants will be destroyed. For a hypothetical case of 10 affected plants, it means that after the plant destruction the grower would lose 490 theoretically healthy and productive plants. Estimating that each plant may produce 1.3 boxes, it means a total of 637 boxes or 3,822.00 USD (estimating 6.00 USD/box at the farm gate). These losses would be recurring yearly becoming the equivalent of permanent since the plantation would be under quarantine and no banana could be planted again to prevent TR4 dispersal.

The size of these zones described in the OIRSA contingency plan were established hypothetically using a large banana monocrop plantation as a model. In practice, the essence of the approach was the procedures associated with the three zones and not the area itself. Thus, the size of the area to be destroyed must be carefully designed on a case-by-case basis. For instance, on the North Coast of Peru, due to the small farm sizes, the grouped layout of fields among multiple owners and the number of affected farms, the measures proposed in the contingency plan were impracticable. Therefore, NPPO implemented only Zone A (**Figure 3C**). This destruction zone is currently limited to two rows of healthy plants on each side of the TR4 outbreak. However, as the pathogen spreads, destruction zones quickly overlap, and growers may lose their banana farms.

The technical aspects of plant destruction and quarantine in first TR4 outbreaks are well established and are mandatory, but these are not always accompanied by comprehensive support that provides credibility, trust, and alternative economical solutions for those farmers losing plants, productive land and in some cases the whole farm. Land use of TR4-infested fields compatible with containment is largely unaddressed. Alternative crops with weekly income like banana are uncommon. In Peru some fields have already returned to rice production, although there are no accompanying strategies to minimize soil movement into drainage ditches or through human or machine traffic.

### Biosecurity

Biosecurity encompasses all the measures to protect society and its members from the negative effects of pest and disease spread (Waage and Mumford, 2008). Biosecurity, just as any other management practice for TR4, should be based on the epidemiology of the disease. Factors and pathways of pathogen dispersal need to be understood using site-specific approaches. Even in the presence of extreme weather factors, biosecurity strategies and measures need to be rigorously implemented and monitored. Regardless of the size of the farm and/or the economic condition of the producer, biosecurity principles should be part of the daily work on the farms.

The effectiveness of biosecurity measures at the farm level depends on several factors that could be grouped into biophysical and socioeconomics. Biophysical factors can be further divided into controllable and uncontrollable.

#### Controllable biophysical factors

Flood irrigation used in many banana cultivation areas not only disperses Foc but can also predispose plants to FWB. In Peru, flood irrigation is essential to maintain banana production. With the arrival of TR4 (Acuña et al., 2022), this practice has become one of the main pathways for pathogen dissemination. Flood irrigation is carried out in many other production areas in Latin America and the Caribbean such as the Dominican Republic, which is still a TR4-free. Growers already organized in irrigation committees need to consider together this factor in advance to prevent the entry and pathogen spread in their farms. Keeping water reservoirs free of pathogens and isolating irrigation from unaffected areas is key. Another controllable biophysical factor is corridors of susceptible host that can facilitate plant-to-plant dispersion. Establishing buffer areas with non-host crops between affected and disease-free areas can reduce pathogen dispersion.

#### Uncontrollable biophysical factors

Floods caused by heavy rains are very common in most banana-producing areas. These events have an immediate direct impact on plantations, destroying them in many cases. However, a rarely considered effect is the impact on the dissemination of pathogens, mainly soil-borne pathogens such as Foc. Heavy rains can transport soil, plant debris, and reproductive structures of pathogens from affected areas to pathogen-free areas. In Colombia, floods that occurred in areas affected by TR4 in the banana zone of Magdalena have been pointed out as the main factor by which the pathogen has spread to disease-free plantations. Similarly, floods caused by the Yaku cyclone have raised the alert about their impact on the TR4 dissemination in northern Peru.

#### Socioeconomic factors

Although some biophysical factors may be difficult to control, socioeconomic factors, where daily activities on farms are grouped, such as shared harvesting and packing crews, could be controllable through the implementation of biosecurity measures. Controlling the movement of these crews with clean in and clean out strategies for footwear, vehicles and tools, single access points among others may be possible is a better coordination is in place. Unfortunately, despite the threat of TR4, only few farms in LAC (considering the total numbers of producers) have implemented biosecurity systems. The lack of implementation of biosecurity measures may depend not only on their cost/ha, but also labor organization and day labor practices, the already intensive current crop management routines with weekly container goals, and the contiguous layout of plots and farms with no buffer zones.

#### On-farm biosecurity: large plantations versus small farms

Australia’s “large farm model” for banana production biosecurity (Kukulies and Velvers, 2017) has been adapted and promoted in Colombia and many other large export banana plantations. This model aims to control and minimize the movement of potential disease vectors across farm boundaries by implementing three zones with increasing access restrictions: farm entrance, reception of off-farm inputs and product processing, and crop production itself. The model focuses on four key elements: 1) Physical barriers: Perimeter fences and ditches prevent the movement of humans, other mammals, and water flow; 2) Access control: A single entrance and exit point allows for tighter control of who and what enters the farm; 3) Biosecurity measures: Footwear, vehicles, and machinery are washed and disinfected at entry and exit points into and out of each zone to minimize contamination and 4) Awareness and education: Clear signage and training for all personnel and visitors educate them about disease symptoms, biosecurity principles, the impact of TR4 on the farm’s income and employment, and potential harm to neighboring farms.

While effective for large banana export farms, these biosecurity measures may be impractical for most global banana production areas. Implementing a “total exclusion” model can be expensive per hectare, making it difficult for small growers to manage. Investing in fences, ditches, walls, and footbaths solely to prevent a potentially non-existent pathogens like TR4 is unattractive for growers already struggling with insufficient capital for basic needs like fertilizers. A study by Staver et al. (2023) in Peru highlighted this challenge. Following the TR4 outbreak in Colombia, researchers assessed biosecurity implementation in five organic banana export associations. Despite introductory training on disease symptoms and transmission, they found virtually no biosecurity measures in place. Mobile packing crews, which move from different farms, harvesting and packing banana from multiple growers periodically, lacked basic sanitation protocols like footwear or vehicle disinfection. Similar limitations were observed in seven banana-coffee farms in Central Peru. While familiar with FWB symptoms, all but one farm had R1 present, forcing three to switch to Cavendish varieties. This demonstrates the challenges smallholders face in implementing extensive biosecurity measures.

Small-scale banana growers face unique biosecurity challenges beyond their limited land area. Sharing resources like access roads, packing centers, irrigation systems, equipment, and even day laborers with tools across multiple farms presents significant risks for pathogen spread. Since day laborers often move between farms, implementing biosecurity measures on individual plots becomes incredibly complex, if not impossible. There is a need for innovative, collaborative biosecurity strategies developed among both growers and epidemiologists. These strategies should consider the specific biophysical and socioeconomic conditions of producers and production zone. One promising approach is “risk mapping”, which can be applied at both the farm level and across multiple farms or small watersheds. This risk assessment could then guide the implementation of essential biosecurity measures in a step-by-step manner. Initial efforts would focus on phytosanitary education to empower growers with knowledge about key factors that spread pathogens. This includes practices like avoiding the exchange of infected planting materials and contaminated soil or water from neighboring farms. Additionally, community-based approaches combined with in-depth socioeconomic studies could aid in implementing tailored biosecurity measures and minimizing pathogen spread.

### Disinfectants

Recent years have seen a significant focus on disinfectant use in biosecurity measures, implemented at border, airports, and by large farms at various entry points (for inputs, banana processing, transportation, and plantations). The effectiveness of disinfectants has been tested in numerous trials (**Table 2**). Studies conducted without the presence of soil have shown that all Quaternary Ammonium Compounds (QACs) are effective against TR4 (Nguyen et al., 2019; Izquierdo-García et al., 2021; Salacinas et al., 2022; Arango-Palacio et al., 2023). Notably, didecyl dimethyl ammonium chloride, 10%-30% (DDAC) and benzalkonium chloride 10% - 25% (BC) disinfectants at a 1% concentration were able to eliminate spores of both TR4 and R1 (Nguyen et al., 2019). Additionally, a glutaraldehyde-based disinfectant also demonstrated efficacy against TR4 (Izquierdo-García et al., 2021).

**Table 2:** Disinfectant with proven effectiveness against *Fusarium oxysporum* f. sp. *cubense*.

Active ingredients tested	Foc Race	Country	Reference
Didecyldimethyl ammonium chloride (10-30%)	R1, TR4	Australia	Nguyen et al., 2019
Benzalkonium chloride (10-25%)			
Alkyl benzyl dimethyl ammonium chloride (10-30%)			
Glutaraldehide, Benzalkonium chloride (20%)	TR4	Colombia	Izquierdo-García et al., 2021
Didecyldimethyl ammonium chloride (12%)			
Benzalkonium chloride +DDAC (12%)			
Di(octyl/decyl) chloride + dimethyl ammonium + benzalkonium chloride (12%)			
Polydimethylsiloxanepolymethacrylate block copolymers containing quaternary ammonium salts	TR4	China	Chang et al., 2021
PQD-BC poly (methacrylamidopropyl benzyl dimethyl ammonium chloride)			
Benzalkonium chloride			
Benzalkonium chloride (40%)	TR4	The Philippines	Salacinas et al., 2022
Di-C8-10-alkyldimethylchlroides + alkyl dimethyl benzyl ammonium chloride (48 + 32%)			
Didecyl dimethyl ammonium chloride (80%)			
Synthesis of amphiphilic polysiloxanes primary ammonium salts (5KDa)	TR4	China	He et al., 2023
Fluorine-containing amphiphilic quaternary ammonium salts	TR4	China	Lin et al., 2023
Didecyldimethylammonium chloride (12%) Alkyldimethylbenzylammonium chloride 80% Di(octyl/decyl) chloride 1% + Benzalkonium chloride 13,5% Di(octyl/decyl) chloride 0,4% + Benzalkonium chloride 1% Quaternary ammonium mixture 10%	R1, TR4	Colombia	Arango-Palacio et al., 2023
Polyacrylamide containing quaternary ammonium salts (PAM-X)	TR4	China	Zhang et al., 2024

The practicalities of disinfectant use in biosecurity have been worked out primarily on large farms. While research confirms the effectiveness of certain disinfectants against TR4, variations exist in factors like dose, exposure time, product concentration, formulation, and commercial name. Users should carefully verify these details and potentially conduct on-site testing to determine the most suitable products and procedures for their specific context. A common challenge encountered with footbaths is the timely replacement of the disinfectant solution, as soil and organic matter compromise its effectiveness. Utilizing indicator strips to measure disinfectant concentration can be helpful. Reactivating the solution to reach the recommended working concentration (usually 1,200 ppm) requires special attention as it can rapidly decrease within 6-12 hours (Lindsay, 2018).

For the daily use of disinfectant, cost becomes an important consideration. Only two studies to identify the most cost-effective products available have been conducted in LAC (**Table 2**). More research is needed to identify affordable and effective disinfectants for the region. Studies with Foc R1, prevalent in many TR4-free countries, could serve as a model for pre-emptive research on disinfectants and other management measures (Izquierdo-García et al., 2021; Arango-Palacio et al., 2023).

However, although QACs are widely recommended and used as disinfectants, potential adverse effects on both human health and the environment needs close attention. These concerns include acute and chronic toxicity for susceptible aquatic organisms, as well as dermal and respiratory effects on humans (Arnold et al., 2023). Additionally, research has demonstrated the potential role of QACs in contributing to antimicrobial resistance (Loughlin et al., 2002).

Beyond the management of disinfectants, the design and setup of footbaths is crucial to reducing the risk of pathogen movement. The presence of soil significantly reduces the effectiveness of disinfectants. Therefore, thoroughly removing soil adhering to shoes and equipment before disinfection is essential. However, both the water and soil generated from footwear cleaning and discarded liquids from footbaths may become a pathogen spreading factors. Secure disposal of soil and wastewater must be guaranteed, an aspect more easily addressed through constructed drains in large farms than on small farms. Even a simple measure like designated footwear only within production areas is not widely practiced on small farms.

## Host resistance

Undoubtedly, the use of resistant varieties is the best strategy for managing FWB. The importance of developing and adopting TR4-resistant varieties is emphasized by the fact that a delay of just five years in adopting such varieties could result in estimated losses of up to 94 billion dollars (Silva et al., 2023). However, fully TR4-resistant genotypes to replace Cavendish or other susceptible commercial varieties are not yet available. Nevertheless, recent progress has been made in phenotyping varieties (Chen et al., 2019; Mintoff et al., 2021; Zhan et al., 2022), evaluating Cavendish tolerant somaclones (Viljoen et al., 2020), and understanding resistance mechanisms (Xie et al., 2023; Zhou et al., 2023).

One aspect that stands out when analyzing research on evaluating TR4 resistance under different conditions is the divergence in protocols used and results obtained by different authors (**Table 3**). The published results to date are in some cases contrasting and difficult to compare (Zuo et al., 2018; Viljoen et al., 2020; Mintoff et al., 2021; Zhan et al., 2022).

**Table 3:** Cultivated banana and plantain varieties and their field resistance against *Fusarium oxysporum* f. sp. *cubense*, tropical race 4.

Subgroup (Genome)	Genotype	Type of inoculum	TR4-Reaction	Reference
Cavendish (AAA)	GCTCV 215	Art.	HR/HR	Mintoff et al., 2021
		NC	R	Zuo et al., 2018
	GCTCV 247	Art.	R/R	Mintoff et al., 2021
		NC	R	Zuo et al., 2018
	GCTCV 218	Art.	I/VS	Mintoff et al., 2021
		NC	MR	Viljoen et al., 2020
	GCTCV 106	Art.	S/S	Mintoff et al., 2021
	GCTCV 119	NC	MR	Viljoen et al., 2020
	Williams	Art.	VS/VS	Mintoff et al., 2021
	Grand Nain (Nandi)	NC	S	Viljoen et al., 2020
	Baxi	NC	I	Zuo et al., 2018
		NC	VS/VS	Zhan et al., 2022
	ZJ 03	NC	MR	Zuo et al., 2018
	ZJ 04	NC	R	Zuo et al., 2018
	ZJ 06	NC	MR	Zuo et al., 2018
Plantain (False Horn, AAB)	Big Ebanga	NC	HR/HR	Zhan et al., 2022
	CB5	NC	HR/HR	Zhan et al., 2022
	CEMSA3/4	NC	HR/HR	Zhan et al., 2022
	Curare enano	NC	R	Zuo et al., 2018
		NC	HR/HR	Zhan et al., 2022
	Kakira	NC	HR/HR	Zhan et al., 2022
	Orishele	NC	HR	Zuo et al., 2018
		NC	HR/R	Zhan et al., 2022
	Batard	NC	HR/HR	Zhan et al., 2022
		NC	HR/HR	Xie et al., 2023
	Batard 2	NC	HR/HR	Zhan et al., 2022
	Plantain N°3	NC	HR/HR	Zhan et al., 2022
Plantain (French, AAB)	Akpakpak	NC	HR	Zuo et al., 2018
	French Plantain	NC	HR/HR	Zhan et al., 2022
	French Sombre	NC	HR/R	Zhan et al., 2022
	Nakatansese	NC	HR/HR	Zhan et al., 2022
	Njombe N◦2	NC	R/I	Zhan et al., 2022
	Ntanga 4	NC	HR/HR	Zhan et al., 2022
	Obino l’Ewai	NC	R	Zuo et al., 2018
	Obubit Ntanga green mutant	NC	R	Zuo et al., 2018
		NC	HR/HR	Zhan et al., 2022
	Uganda Plantain	NC	I/S	Zhan et al., 2022
	Ihitisim	NC	HR/HR	Zhan et al., 2022
Iholena (AAB)	Kofi	NC	HR/HR	Zhan et al., 2022
	Luba	NC	S/VS	Zhan et al., 2022
	Maritú	NC	I/S	Zhan et al., 2022
	Rukumamb	NC	R/I	Zhan et al., 2022
	Tigua	NC	S/VS	Zhan et al., 2022
	Uzakan	NC	S/VS	Zhan et al., 2022
	Wisu	NC	VS/VS	Zhan et al., 2022
Maoli Popoulu (AAB)	Pacific Plantain	NC	VS/VS	Zhan et al., 2022
	Poingo	NC	R	Zuo et al., 2018
		NC	HR/HR	Zhan et al., 2022
Mysore (AAB)	Pisang Ceylan	NC	I/I	Zhan et al., 2022
	Thap Maeo	NC	R	Zuo et al., 2018
Ney Mannan (ABB)	Blue Java	NC	R	Zuo et al., 2018
Pisang Awak (ABB)	Dwarf Ducasse	Art.	S/VS	Mintoff et al., 2021
	Guangfen No. 1	NC	S	Zuo et al., 2018
	Namwa Khom	NC	R	Zuo et al., 2018
Pisang Raja (AAB)	Pisang Raja Bulu	NC	S/VS	Zhan et al., 2022
	Pisang Raja N°2	NC	I/I	Zhan et al., 2022
	Pisang Rajah	NC	I/S	Zhan et al., 2022
	YN2	NC	HR/R	Zhan et al., 2022
	Pisang Raja	NC	HR/HR	Zhan et al., 2022
Silk (AAB)	Amrithapani	NC	VS/VS	Zhan et al., 2022
	Digjowa	NC	VS/VS	Zhan et al., 2022
	Figue Pomme Géante	NC	VS/VS	Zhan et al., 2022
	Malbhog	NC	S/S	Zhan et al., 2022
	Silk	NC	VS/VS	Xie et al., 2023
Hybrids	FHIA-01 (AAAB)	Art.	HR/HR	Mintoff et al., 2021
	FHIA-02 (AAAA)	Art.	HR/HR	Mintoff et al., 2021
	FHIA-03(AABB)	Art.	R/I	Mintoff et al., 2021
	FHIA-18 (AAAB)	Art.*	HR/R	Mintoff et al., 2021
	FHIA-21 (AAAB)	NC	R	Zuo et al., 2018
		NC	HR/HR	Zhan et al., 2022
	FHIA-25 (AAAA)	Art.	HR/HR	Mintoff et al., 2021
	FHIA-26 (AAAA)	NC	R	Zuo et al., 2018

Art, Artificial inoculation by using inoculum produced in the laboratory; NC, Natural conditions (infested soils).

HR, Highly Resistant; R, Resistant; I, Intermediate; MR, Moderately Resistant; S, Susceptible; VS, Very susceptible; HS, Highly susceptible.

The somaclonal variant GCTCV-218 (‘Formosana’) is a case to highlight due to its commercial importance. Promoted as a TR4-tolerant genotype, but also because it is a Cavendish type, GCTCV-218 has replaced traditional Cavendish cultivars, such as Grand Naine and William in TR4-affected areas in the Philippines and Mozambique (Viljoen et al., 2020). In experiments conducted under natural conditions of TR4 infection in Mozambique, Viljoen et al. (2020) ranked GCTCV-218 as moderately resistant. Interestingly, Mintoff et al. (2021) classified GCTCV-218 as intermediately resistant in the first cropping cycle and highly susceptible in the second one. Factors such as inoculum pressure and type of inoculation may have interfered with these results (**Table 3**). Understanding the inoculum pressure levels that GCTCV-218 (‘Formosana’) is capable of withstanding and the biotic and abiotic factors affecting its resistance to TR4 is key to guiding producers in growing this variety in TR4-affected fields and extending its useful life.

Another subgroup that should be carefully considered in evaluating resistance to TR4 is plantains (AAB). This subgroup is crucial for food security in many LAC countries, where around 1 million hectares are harvested every year. So far, out of the 19 plantain genotypes evaluated for TR4 resistance in field conditions, most have been classified as resistant or highly resistant (**Table 3**). Only two varieties (‘Njombe N◦2’ and ‘Uganda Plantain’) have been classified as intermediate and susceptible, respectively (Zhan et al., 2022).The varieties Curare Enano and CEMSA3/4, which are of high importance for LAC, have been classified as resistant or highly resistant (Zuo et al., 2018; Zhan et al., 2022). Interestingly, ‘Curare Enano’ was ranked as resistant by Zuo et al. (2018) and as highly resistant by Zhan et al. (2022). These divergences might be related to the degree of TR4 infection observed in this variety under greenhouse conditions.

However, it is important to note that these studies were conducted in China under different soil and climatic conditions than those found in LAC. Furthermore, the genotypes evaluated in these studies may not represent the existing intra-varietal variability present in LAC. Therefore, evaluate the main plantains varieties present in LAC under field conditions in the region is critical. This evaluation can be conducted in affected areas in Colombia, Peru, and/or Venezuela, where TR4 is present, and plantains are essential for food security.

In addition, other subgroups, such as Iholena, Maoli Popoulu, Mysore, Ney Mannan, Pisang Awak, Pisang Raja and Silk, are important for local markets, mainly as dessert bananas. These subgroups have also been evaluated for TR4 resistance and only eight out of the 26 varieties evaluated have been ranked as resistant or highly resistant (**Table 3**). Although many of these varieties were already susceptible to Foc R1, the wide host range of TR4 reinforces the claim that TR4 is not just a Cavendish issue. Field studies comparing the aggressiveness of R1 and TR4 on these varieties have not been conducted, but most of these varieties showed disease index even higher than Cavendish (Zhan et al., 2022). Therefore, growers who are used to dealing with R1 may face even more significant challenges when dealing with TR4. As a result, breeding efforts for TR4 resistance should not exclusively target Cavendish but also consider other AAB subgroups.

It is well-known that protocols used to discriminate the resistance levels of any host are crucial. However, for soil-borne pathogens such as Foc and for host-pathogen interactions where the genetic basis of disease resistance is poorly understood, as is the case of banana-Foc, this aspect is critical. Early screening for resistant genotypes under greenhouse conditions can potentially identify promising genotypes much more rapidly than field phenotyping and generate knowledge about the genetic and molecular basis of resistance. However, greenhouse screening is performed under artificial conditions, which often include high inoculum levels, young plants, autoclaved potting media, and controlled environmental conditions, and in some case might not reflect field conditions. This is particularly relevant from the studies conducted by Zuo et al. (2018) and Zhan et al. (2022) where most of the varieties showed some degree of TR4 infection (intermediate resistance) in the greenhouse, but later in the field, they were ranked as resistant or highly resistant (**Table 3**). Such was the case for 13 plantains varieties. Defining the correlation between greenhouse and field studies is crucial for the selection process of new varieties and determining whether a given variety is resistant or susceptible. This is especially critical for varieties of high economic importance, such as plantains, as it may support risk analysis and guide management strategies at different levels.

Accurately assessing banana varieties’ resistance to FWB requires either estimating the pathogen density in the test area or using a controlled inoculum concentration. While technically feasible, directly measuring the pathogen in soil presents challenges related to sampling, soil type, scaling up the process, and costs (Matthews et al., 2020). Evaluating varieties across at least two cropping cycles in well-characterized soil (considering both biotic and abiotic factors) is recommended.

Knowledge about the banana - TR4 interactions has been expanded in recent years and numerous genes have been found associated with the defense response process. Efforts have been focused not only on identifying resistant banana varieties but also on unraveling the mechanisms underlying their resistance to TR4. However, as expected most the research has been conducted in controlled environments such as greenhouses, which limits our ability to address questions crucial for field management strategies.

It is widely acknowledged that mother plants transmit Foc to subsequent generations (suckers), but are defense responses primed in mother plants also induced to suckers? Is a treatment that triggers resistance genes in the mother plant equally effective in the suckers? What specific defense genes are activated in the suckers, and for how long do these responses persist? Furthermore, what factors contribute to the gradual loss of resistance in the ratoon, potentially altering the resistance dynamics over time? Moreover, is the mother plant capable of “tolerating” the initial inoculum in the soil via pre-formed defense responses (cell wall reinforcement, lignin deposition, etc.) and induced defense responses, and therefore this reaction is not present to guarantee the resistance when the inoculum is transmitted from mother to suckers through the vascular system? These critical questions remain unanswered, highlighting the need for further investigation into the intricate dynamics of banana-TR4 interactions in field conditions. Some of these answers could help not only breeding programs but also to understand and manage in the field the behavior of varieties that change from resistant to susceptible after the first crop cycle (**Table 3**; Mintoff et al., 2021; Zhang et al., 2022). A deeper comprehension of the correlation between the host’s response timing to the pathogen, symptom expression, and inoculum pressure is essential. This understanding is not only crucial for categorizing specific varieties as resistant or susceptible but also for guiding management practices, particularly for varieties with intermediary resistance, such as somaclonal variants (e.g., GCTCV-218).

To date, conventional breeding efforts have not yielded varieties resistant to TR4 or any other disease resistance that meet export market demands. The somaclonal variants tested have exhibited only intermediary resistance (**Table 3**). The development of the science of transgenic or genetically edited plants applied in banana (Dale et al., 2017) offers a new path to improved varieties. Notably, the Australian government recently granted approval for the commercialization of the genetically modified Cavendish variety “QCav4” resistant to TR4 (Burton, 2024). While this marks progress in regulatory terms within Australia, the logic of widespread conversion to this new variety on-farm remains to be tested. The production and export of such varieties in LAC will also encounter important challenges: 1) varying authorization processes for field validation tests across different countries; 2) time lags from field validation to commercial production permits; and 3) conventional and organic acceptance from importing countries, such as those from the European Union. Uptake of resistant varieties is likely to be highly selective in response to disease spread for a sector (and region). In over 100 years of banana export, a change in varieties occurred only once when R1 became widespread in the 1960s. Small-scale producers for domestic markets, while operating with more variety flexibility, are highly dependent on low-cost planting material from informal sources. A shift to tissue-culture planting material would represent a major cost and logistical transition.

## Biological control

Recently, Bubici et al. (2019) published a comprehensive review of the potential of biological control against FWB. Since then, numerous research papers have been published exploring different strategies for the biological management of FWB using beneficial fungi and bacteria alone or in combination. However, robust field-tested studies using biological control agents (BCAs) to manage FWB which are summarized in **Table 4** are not numerous.

**Table 4:** Biocontrol agents and their effectiveness against Fusarium wilt of banana^*^.

Microorganisms (Strain)	Observed effects	Country	Genotype	Foc race	Reference
*Bacillus subtilis* (EPB56 and EPB10) and *Pseudomonas fluorescens* (Pf1)	Reduced FWB incidence by 78% in treated plants compared to untreated control.	India	Red Banana (AAA)	R1	Kavino and Manoranjitham, 2018
*Trichoderma reesei* (CSR T-3) and *Lysnibacillus fusiformis* (CSR-A-11)	A formulation of *T. reesei* and *L. fusiformis* reduced FWB incidence by 40%. Incidence on treated plants: 6,08%. Incidence on untreated plants: 45.68%.	India	Cavendish (AAA)	TR4	Damodaran et al., 2019
Actinomycetes (AQ6, AQ3, and AQ121)	Reduced FWB incidence by 18%. Treated plants: 18.33%. Control: 36.67%.	Philippines	Cavendish (AAA)	TR4	Paulite, 2019
*Trichoderma reesei* (CSR-T-3)	Reduced FWB incidence. Treated plants: 10.58%. Control: 46.50%.	India	Cavendish (AAA)	TR4	Damodaran et al., 2020
*Bacillus mycoides* (NP02) and *Bacillus amyloliquefaciens* (BaPD1)	*B. amyloliquefaciens* increased the survival rate of banana seedlings reducing the disease severity index by 1.45-fold. *B. mycoides* showed significant plant growth promotion.	Taiwan	Cavendish (AAA)	TR4	Lin and Ho, 2021
*Paenibacillus terrae* (Pt05), *Burkholderia cepacia* (Bc11), *Bacillus amyloliquefaciens* (Ba62) and *Trichoderma harzianum* (gz-2)	Field applications of strain combinations showed 57.14% control of FWB. A reference for combining biocontrol agents by using phylogenetic data is provided.	China	Cavendish (AAA), Cv. Williams	TR4	Du et al., 2022
*Pseudomonas fluorescens; Trichoderma viride* and *Paecilomyces lilacinus*	Reduced incidence on FWB. Plants treated with *P. fluorescens* (at planting, 2nd, 4th, and 6th month after planting): 10.43%. Plants treated with *T. viride + P. lilacinus* (each at 10 g/plant as basal, 2nd, 4th, and 6th month after planting): 8.25%. Control: 29.82%.	India	Cavendish (AAA)	R1	Jasmine et al., 2022
*Piriformospore indica* and *Streptomyces morookaensis* (4-1986)	Reduced FWB incidence. Treated plants: 11.7%. Control plants:100%	China	Cavendish (AAA)	TR4	Zhu et al., 2023

^*^Despite many valuable works that have been carried out in vitro and under greenhouse conditions this review made more emphasis on results from field experiments.

*Trichoderma* spp. is by far the most studied fungi for controlling FWB (Bubici et al., 2019; Damodaran et al., 2020; Du et al., 2022; Zhang et al., 2022). Being a multipurpose microorganism, *Trichoderma* spp., utilizes direct antagonism and competition in the rhizosphere, colonizes plant roots (externally and/or as an endophyte), and has the capacity to promote plant growth and prime local and systemic defense responses against biotic stresses. These attributes make *Trichoderma* spp. an excellent option for managing a soil-borne pathogen, such as Foc (Woo et al., 2023).

Several bacteria, including *Bacillus* spp., *Pseudomonas* spp., *Streptomyces* spp., and *Burkholderia* spp., have been studied in the greenhouse and in field conditions with promising results against FWB (Kavino and Manoranjitham, 2018; Fan et al., 2021; Zhang et al., 2021; Cao et al., 2022).

The combined use of *Trichoderma* and beneficial bacteria to develop microbial bioinoculants with synergistic activities has gained more attention (Damodaran et al., 2019; Jasmine et al., 2022; Poveda and Eugui, 2022). The approach referred to as synthetic microbial communities was applied by Prigigallo et al. (2022). They tested combinations of 44, 12 and 3 isolates in a mixture. They propose to continue research with more complex combinations, but the 3-isolate mixture was promising enough in the greenhouse to move to the field. In this section and the following on integrated field management, mixtures are often among the treatments tested.

In India, the world’s largest banana producer, where TR4 was officially reported in 2018 (Damodaran et al., 2018), the efficacy of *Trichoderma reesei* (CSR-T-3) in reducing the incidence of FWB and TR4 has been demonstrated alone (Damodaran et al., 2020) and in combination with the bacteria *Lysnibacillus fusiformis*, strain CSR-A-11 (Damodaran et al., 2019). Additionally (Damodaran et al., 2020), showed that *Trichoderma reesei* (CSR-T-3) can promote banana plant growth and improve yield indices.

In China, where Foc TR4 is widely distributed and has devastated thousands of hectares of bananas, biocontrol strategies based on the combination of microorganisms have also been explored with encouraging results under field conditions (Lin and Ho, 2021; Du et al., 2022; Zhu et al., 2023).

Different strains of *Bacillus* spp. and *Pseudomonas* spp. have been studied against FWB under field conditions, and actinomycetes, especially *Streptomyces* spp., are gaining increasing attention (Paulite and Paulite, 2019; Zhu et al., 2023). In fact, the combined treatment with the endophytic fungus *Piriformospore indica* and the rhizospheric bacterium *Streptomyces morookaensis* (strain 4-1986) significantly reduced FWB in field plots highly affected by FWB TR4 (Zhu et al., 2023). The authors conducted field trials for two consecutive years in a field that had been abandoned due to severe Foc TR4 infestation. Seven days before field transplantation, the soil and banana plantlets were treated with *S. morookaensis* (4-1986) and *P. indica* (a root-colonizing endophytic fungus). After the first cropping cycle, the incidence of FWB on treated plants was 11.7%. Control (untreated) plants showed 100% incidence of FWB. Banana plants exhibiting FWB symptoms in the first cycle in the treated plot were cut and treated again with *S. morookaensis* (Sm4-1986). After eight months, the incidence of FWB in the previously infected banana mats re-treated with *S. morookaensis* (Sm4-1986) was reduced to 9.1%, indicating a curative effect of *S. morookaensis*. These results demonstrate that the combination of the fungi *P. indica* and actinomycete *S. morookaensis* reduced FWB TR4 in a heavily infested field. Notably, the authors also reported that infected mats were recovered by re-applying *S*. *morookaensis*. While recovering TR4-infected mats is promising, these observations should be approached with caution as this phenomenon is also observed in banana commercial fields without any treatment.

The biocontrol of FWB in the field continues to be a challenge, not only because of the disease’s epidemiology (Pegg et al., 2019) but also due to perennial nature of banana and inherent characteristics of banana production systems (Dita et al., 2018). Consequently, the biocontrol of FWB, as with many other pathosystems, should not be seen as a tool to be used in isolation but rather implemented in an integrated management system (Dita et al., 2018; Bubici et al., 2019).

Biocontrol strategies to manage FWB in the field could also benefit from innovative approaches, such as using banana root exudates to enhance the colonization efficiency of BCAs through chemotaxis (Yang et al., 2022) or adding antifungal compounds, such as iturin A5 produced by thermotolerant marine *Bacillus amyloliquefaciens* (Singh et al., 2021). Moreover, the use of biochar, a solid compound produced by the decomposition of organic matter at high temperatures and low oxygen concentration in a pyrolysis process, to deliver BCAs in bananas to manage FWB, also deserves attention (Din et al., 2018). Biochar can improve soil physical, chemical, and biological parameters alone and potentiate the activities of BCAs (Iacomino et al., 2022).The high carbon content in biochar and its ability to remain in the soil for long periods make it an excellent substrate source for microorganisms. Additionally, it offers opportunities to serve as an inoculant vehicle for BCAs (Iacomino et al., 2022).

Research papers showing positive results of BCAs to manage FWB under field conditions are still scarce. However, studies using BCAs as the only management alternative have reported high control levels of FWB under field conditions. Unfortunately, data on the chemical, physical, and biological characteristics of these soils, as well as the inoculum densities of the pathogen in the soil, are rarely presented.

The successful formulation and delivery of a microbial inoculant do not necessarily ensure increased crop productivity, as there are several environmental and microbial factors that can impact its efficacy in the field. Therefore, the effectiveness of applying BCAs to manage FWB may depend on the production systems used. Some companies deliver these microorganisms through irrigation systems, but this is not feasible for most producers in LAC who do not use irrigation in their farms. In such cases, BCAs may need to be applied in conjunction with other procedures, such as the use of organic fertilizers or amendments like biochar. For smallholders, challenges may be even greater, including the need for proper storage and preparation of biological product mixtures and affordable methods for field application.

Research is still primarily at the stage of testing the biological potential for FWB management. For instance, field studies on the use of BCAs against FFWB in LAC are scarce. Finding practical applications will represent many additional challenges which need to be addressed such as a) Identification of appropriate organisms may need to be country-specific guided by research results in other countries, since micro-organisms are considered part of biodiversity belonging to each country; b) Application method and frequency: in irrigation water with drip or sprinkler; incorporated into biofertilizers; use with biochar; varied methods depending on crop stage – pre-planting, planting, established plantation; c) Use of combined practices like banana root exudates and antifungal substances and d) Management of other practices in the production system which may negatively affect biological control agents such as fungicides for leaf disease management.

## Integrated soil health-oriented practices and cropping systems

The components for viable banana cropping systems in the presence of TR4 documented in the previous sections –resistant varieties and biological control agents – indicate that recovery of banana production in zones affected by TR4 might be on the horizon. However, market-acceptable varietal substitution like Cavendish for R1 may be much farther in the future. More likely are site-specific integrated cropping systems based on multiple factors contributing to disease suppression and greater plant vigor. In Peru, many growers in affected farms have shifted to other crops, although in Taiwan, China and Philippines growers use rest periods, tolerant varieties, and microbial products to recover banana production. The challenge to recover banana production in TR4-affected lands will only grow as the disease spreads.

Different biotic and abiotic factors interact to generate either more conducive or suppressive conditions for banana production. The soil and the cropping system are a dynamic ecosystem teeming with complex interactions that can significantly impact the prevalence and severity of FWB and banana production. Here we review recent advances in our understanding of soil chemical and physical properties and the use of amendments and fertilizers and crop diversification to limit the disease in Foc-infected soils.

### Soil chemical and physical properties

Soil pH is the indicator most associated to FWB as acid soils have been frequently associated with severe disease expression on both Foc R1 (Segura et al., 2021; Teixeira et al., 2021)) and Foc TR4 interactions (Rodríguez-Yzquierdo et al., 2023).

Orr and Nelson (2018) reviewed studies on Fusarium wilts for all horticultural crops, including banana. They identified an inverse correlation between pH levels and disease severity across various crops in 21 studies. However, they emphasized the importance of caution, noting that pH adjustments can influence both microbial populations and cation availability, which need to be monitored when adjusting pH through inputs.

It has been found that raising pH to levels still favorable for bananas, but not for Foc could be effective to reduce FWB (Dita et al., 2018; Pegg et al., 2019) However, soils with low acidity (pH above 7) do not guarantee suppressiveness or low FWB. Heavily affected banana plantations by TR4 in northern Peru and Venezuela have soil pH even higher than 7.5 (Rodríguez-Yzquierdo et al., 2023). Furthermore, none of the experiments in **Table 5** adjusted pH as part of the treatments.

**Table 5:** Management practices and their effectiveness against *Fusarium* wilt of banana under field conditions.

Practice	Observed effects	Country	Variety (Genome)	Foc race	Reference
Crop rotation (Pineapple)	FWB incidence in the pineapple - banana rotation treatment was significantly lower (30%) than that the monoculture treatment (>60%). Pineapple rotation significantly decreased TR4 population in the soil.	China	Cavendish (AAA)	TR4	Yuan et al., 2021
Use of biofertilizer and crop rotation (Pineapple)	Rotating pineapple and banana combined with biofertilizer application showed the lowest FWB incidence (12.3%). Rotating pineapple and banana with organic regular fertilization showed 33.3% of incidence. The highest FWB incidence (66.8%) was observed in banana monoculture with organic fertilizer.	China	Cavendish (AAA)	TR4	Wang et al., 2022
Green manure intercropping (*Crotalaria acicularis, Sesbania sesban, Melilotus officinalis, Vicia villosa and Trifolium repens*)	FWB incidence on intercropped treatments was reduced (26,92 – 40,77%) when compared with non-intercropping (> 60%). *V. villosa* and *T. repens* intercropping treatments showed greater effect in disease suppression than the other treatments.	China	Cavendish (AAA)	TR4	Yang et al., 2022
Fertilization (N, Ca Mg and Mn)	No effect of mineral fertilization on FWB incidence was observed. Reduced plant mortality was associated with soil acidity.	Costa Rica	Gross Michel (AAA)	R1	Segura et al., 2021
Crop rotation (Pepper) and organic fertilizer	Reduced FWB incidence (32%) after chili pepper-banana rotation when compared with banana monocrop (53%).	China	Cavendish (AAA)	TR4	Hong et al., 2020
Combined chemical fertilization (CF) and organic matter application (OM)	Twelve treatments combining CF and OM fertilization were compared to CF only. 50% of CF + 500 g/plant of OM showed highest FWB reduction.	China	Cavendish (AAA)	TR4	Sun et al., 2018
Alternative soil management practices (use of nitric N source, heat-treated rock phosphate, organic compost) compared to conventional soil management	FWB incidence was > 90% higher in the control than in the alternative treatments in the first cropping cycle. Effectiveness of treatments decreased gradually.	Brazil	Prata (Pome AAB)	R1	Teixeira et al., 2022
Crop rotation (pepper, sugarcane, wax gourd, and pumpkin)	All crop rotation decreased FWB incidence when compared with monocropping.	China	Cavendish (AAA)	TR4	Fan et al., 2020
Crop rotation (Pepper and Eggplant) and organic fertilizer or biofertilizer application.	Pepper-banana and eggplant-banana rotation reduced FWB incidence and pathogen abundance. Biofertilizers enhanced the positive effect of crop rotation.	China	Cavendish (AAA)	TR4	Hong et al., 2023
Intercropping (three varieties of Chinese chive)	Intercropping banana with the three varieties of Chinese chive reduced FWB incidence and increased banana yield compared to the monoculture control.	China	Cavendish (AAA)	TR4	Li et al., 2020
Ground covers (*Arachis pintoi*)	Ground cover with *A. pintoi* induced alterations in the soil microbiome and promoted FWB suppression.	Australia	Ducasse (ABB)	R1	Rames et al., 2018
Application of organic fertilizer, chemical fertilizer (NPK), *Bacillus mycoides* (BM) and *Bacillus amyloliquefaciens* (BA)	BM promoted plant growth and BA increased survival rate of banana seedlings. Adequate plant nutrition also enhanced plant resistance to FWB	China	Cavendish (AAA)	TR4	Lin and Ho, 2021
Intercropping (*Trifolium* *repens)*	Intercropping *T. repens* with banana showed 32.78% of FWB incidence while monoculture presented 46.11%	China	Cavendish (AAA)	TR4	Ren et al., 2024
Neem cake (NC) + Farmyard Manure (FYM), *Trichoderma viride* + *Pseudomonas fluorescens*+ *Paecilomyces lilacinus* + Carbofuran + Stem injection of Carbendazim.	Plants treated with NC +FYM + *T. viride* + *P. fluorescens* + *P. lilacinus* + Carbofuran + Carbendazim showed lower FWB incidence (11.12%) than control treatment (88.89%).	India	Ney Poovan (AB)	R1	Murali et al., 2023

Soil physical characteristics promoting vigorous and healthy root growth are generally linked to soil texture and structure (low root penetration resistance, high soil aeration), but results of FWB incidence associated to specific soil textures vary by region (Dita et al., 2018). In Brazil, higher incidence of FWB was observed in plantations with both high soil density and resistance to root penetration (Teixeira et al., 2021).

Soil nutrient levels have been associated with disease severity in different field surveys. The review by Orr and Nelson (2018) of soil chemical properties for Fusarium wilt in different crops found strong agreement for adequate nutrition of several macro and micronutrients - calcium, silicon, potassium, and zinc in pot experiments and field trials. Their review concludes that high levels of available forms of these nutrients should be part of crop management to reduce TR4 impacts. Segura et al. (2021) tested the use of mineral fertilization in soils with contrasting pH and found reduced mat mortality and disease expression with improved chemical properties. However, they concluded as well that soil chemical management alone would not prevent disease buildup.

Higher levels of iron and manganese, on the other hand, were found to favor FWB severity (Orr and Nelson, 2018). However, pot trials to manage the nutrients through chelating to suppress FWB at field scale were unsuccessful (Orr et al., 2021) in soils with high iron levels.

The relationship between soil N and FWB is a critical factor that influences the occurrence, severity, and management of the disease. N is an essential nutrient for plant growth and development, but its availability in the soil can impact the dynamics of Fusarium wilt in several ways (Mur et al., 2016). The N fertilization of banana plants, under certain soil conditions, can impact the intensity of FWB. Whether due to the acidifying effect of ammoniacal sources or their direct impact on plant resistance, they can contribute to an increase in disease severity (Segura et al., 2021; Orr et al., 2022).

When N is applied from ammoniacal sources rather than nitric sources, it tends to favor FWB. The influence of ammonia sources on soil acidification, changes in the microbiome, and their direct impact on the pathogen-host relationship appear to be significant factors contributing to the escalation of FWB (Dong et al., 2015; Mur et al., 2016; Wang et al., 2016). Recent research (Orr et al., 2022) concluded the source may not be as important as the dose. High rates of N fertilizer, and associated increased plant growth, were linked with increases in disease due to a trade-off between growth and defense. The authors propose that N applications should tend more towards nitrates and target plant performance without compromising plant defenses.

### Organic amendments, biofertilizers and diversification of banana monocrops

Soil organic matter greatly influences soil structure, nutrient availability, microbial activity, and overall soil health (Creamer et al., 2022). Due to these multiple roles, the addition of organic material can have implications for the occurrence and severity of FWB (Sun et al., 2018). Cropping systems and soil management oriented towards increasing and diversifying organic inputs are core aspects of induced soil suppressiveness (Mareckova et al., 2023). The experiments in **Table 5** include among treatments compost application, the use of organic materials enriched with specific microbes, intercropping with living ground covers and chives and crop rotations with horticultural crops.

All the reported management options result in a diversification of organic matter inputs compared to a banana monocrop. Few standardized measurements were consistently employed across all experiments to gauge the effects of the treatments. This lack of uniformity makes it challenging to compare results and evaluate their practicality for application in production areas. All the reported trials found a positive treatment effect, although often only momentary. However, a considerable portion of the reviewed experiments concerning organic fertilization exhibits limitations in practical validity. The composition of the organic sources examined is frequently deficient, lacking in detailed information or clarity regarding the process of obtaining them. Organic sources, by nature, exhibit substantial variation in terms of composition, impact on soil chemical attributes, microbiota, and plant responses. Furthermore, the mode of application is occasionally not well-documented, posing challenges in extrapolating results and applying findings to the formulation of orchard management strategies.

Crop diversification included rotations with chili pepper, eggplant, wax gourd, pineapple, and sugar cane, and intercropping with chives and different legumes was tested generally with positive results on FWB symptoms. Crop residue generated and cropping practices applied during the rotation are not always reported. Only three experiments monitored crop performance beyond first harvest. These multi-harvest experiments also followed protocols for the management of infected plants to reduce inoculum, a topic of great relevance for on-going recovery of banana production in TR4 affected soils.

Detailed studies of the experimental treatments through pot trials and molecular analysis uncovered diverse mechanisms for disease suppression which highlight future directions for TR4-suppressive banana cropping systems. Intercropped Chinese chives produced anti-fungal substances which reduced FWB incidence and severity in banana with significant Chinese chive varietal differences in antifungal potential (Li et al., 2020). Pineapple in rotation with banana generated residues for incorporation which promoted fungal organisms responsible both for the secretion of anti-microbial substances and indirect competition for nutrients with FWB (Yuan et al., 2021).

Through the combined application of biofertilizers and pineapple rotations, Wang et al. (2022), emphasized the additive impact of these practices on both fungal and microbial networks, leading to increased Foc suppression. Suppressive effects to FWB were also demonstrated by a crop cycle of pepper or eggplant prior to banana plantation (Hong et al., 2023) and by intercropping *Trifolium repens* (Ren et al., 2024).

In summary, the field trials testing cropping system approaches to the recovery of FWB-affected soils offer many treatment options and field and laboratory methods to track results. Combinations of practices - use of organic amendments + crop diversification + application of specific microbes – showed the most promising results. In the final section we address research challenges in the next phase of cropping systems research. One important component still missing from our review is the reduction of Foc inoculum through the management of infected plants, starting from the first sick plants through to greater than half the plants which is the level cited by the different experimental groups about the history of FWB in their experimental fields.

## Conclusion, lessons, research gaps and priorities for action

**Figure 4:**
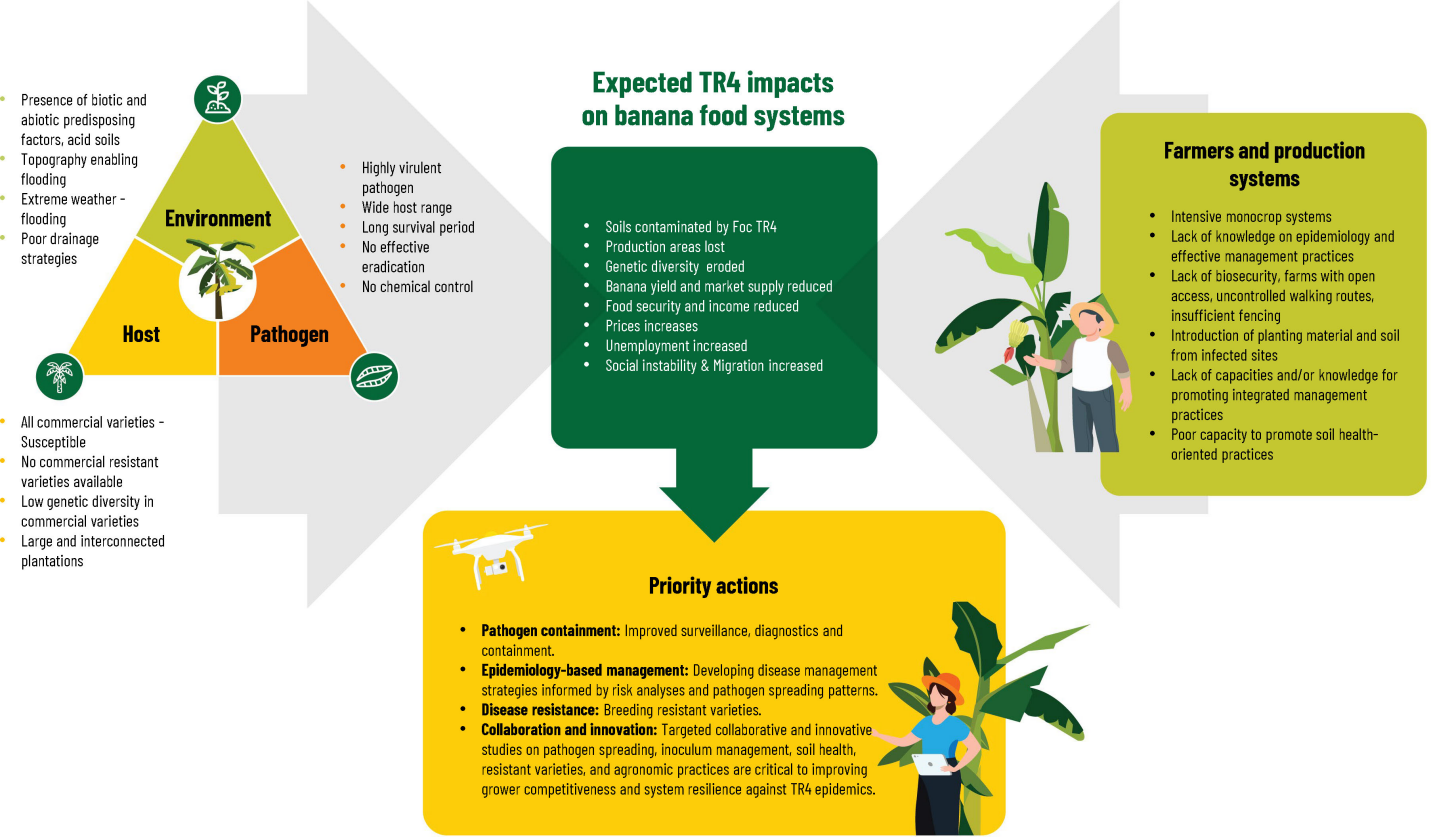
Overview of the current epidemiological (left panel), farmers and production systems (right panel) situations, expected impacts (upper panel) and a priority actions (bottom panel) of Fusarium wilt tropical race 4 (TR4) in Latin America and the Caribbean.

Considering the current epidemiological factors (**Figure 4**, left panel), and the characteristics of farmers and production systems (**Figure 4**, right panel), the TR4 outbreaks in LAC appear to be only the beginning of the inevitable dissemination of the pathogen within already affected banana-producing countries and into new countries. The alarm is justified given the livelihood importance both for export and national consumption of bananas and plantain in LAC and the expected impacts or TR4 dissemination (**Figure 4**, top panel). At the same time in expanding both research and actions, we should consider an important first lesson. LAC still has millions of unaffected banana hectares. In Colombia in spite the disease spread from the initial seven farms reported in 2019 to 20 farms, more than 99% of the planted area of banana (about 50.000 ha) and plantains (about 430.000 ha) of the country is still TR4-free (ICA, 2024). In Peru where the TR4 is restricted to the northern area of Piura, 330 hectares are TR4-infected of the estimated 171 000 banana hectares in the country (FAOSAT, 2022) and many more thousands of hectares suitable for banana production, but in other uses. In Venezuela, TR4 is present in three states, but no information is available of how many hectares are affected from the total of 95.562 ha of banana and plantain planted in the country (INSAI, 2023a). A deliberate and timely response is needed not as an emergency, but as a new and ongoing dimension of banana production systems and the accompanying programs of NPPOs, farm level biosecurity and production practices. Quarantine and biosecurity measures will need to become permanent management components either for exclusion or production in the presence of the pathogen. A second lesson provides us with a framework for thinking about research gaps and priorities for action on two fronts. First, public plant protection programs and farm and value chain biosecurity measures need to incorporate a better understanding of risk analysis and epidemiology for greater efficiency and effectiveness over the long haul (see sections Early Detection and Biosecurity). Second, researchers, growers and input providers will need to focus on integrated production systems which will not only allow banana production in TR4-infected soils, but also address of concerns around banana production sustainability – soil health, water and carbon footprint, externalities from input use and labor productivity.

TR4 is just one of many phytosanitary threats to agriculture and the food system in LAC. Banana stakeholders will need to find synergies and alliances across the agriculture and value chain landscape to strengthen both preventive and reactive responses. Reshaped banana production systems focused on soil health, management of inoculum levels and appropriate agronomic practices may also be less vulnerable to destructive epidemics, such as TR4, but will require targeted studies to maintain grower competitiveness (**Figure 4**, bottom panel). The productive life of moderately susceptible clones can be increased with innovative soil management practices that suppress the pathogen and promote plant defenses (see sections Host Resistance, Biological Control and Integrated Soil Health). A critical factor for recovering Foc-contaminated lands is to reduce inoculum levels in the soil as the disease progresses. Management of soil and cropping systems health in contaminated lands should consider: 1) Early detection and plant destruction; 2) Minimal soil movement and maximized soil cover to reduce the spread of Foc-contaminated soil particles either in routine daily practices or during extreme weather events; 3) Increasing disease-suppressiveness of soils and 4) Crop production strategies to increase productivity with economic efficiency compatible with the reduction of other plant health problems.

General overviews like this review and others cited in the introduction (Pegg et al., 2019; Drenth and Kema, 2021; Silva et al., 2023) provide broad guidance on biosecurity measures and research options, but they may not be tailored to specific countries or territories. This lack of specificity fails to account for differences in institutional and human capacities, as well as disease epidemiology, which vary from one location to another. Each country in LAC backed by international agencies, regional cooperative agreements and advanced national and international banana research programs will need to develop adoptable innovations for a) Pathogen containment through improved surveillance and diagnostic capacities, b) disease-resistant commercial varieties based on breeding, introduction and testing and multiplication of planting material, c) biological control organisms collected, characterized and screened nationally and d) epidemiology-based disease management strategies developed in close collaboration with growers. Our review suggests that efforts have accelerated in recent years, but more needs to be done in key areas we have identified.

## Author contributions

TM: Conceptualization, Methodology, Writing – original draft, Writing – review & editing. JV: Methodology, Validation, Writing – original draft, Writing – review & editing. LT: Conceptualization, Methodology, Writing – original draft, Writing – review & editing. CS: Supervision, Writing – original draft, Writing – review & editing. MD: Conceptualization, Funding acquisition, Project administration, Supervision, Writing – original draft, Writing – review & editing.
